# Voluntary wheel-running activities ameliorate depressive-like behaviors in mouse dry eye models

**DOI:** 10.3389/fnbeh.2022.925128

**Published:** 2022-09-09

**Authors:** Katsuya Nakano, Hitomi Nakazawa, Qiang He, Junsuke Uwada, Takeshi Kiyoi, Takaharu Ishibashi, Takayoshi Masuoka

**Affiliations:** ^1^Department of Pharmacology, School of Medicine, Kanazawa Medical University, Uchinada, Japan; ^2^Clinical Research and Trials Center, Kanazawa Medical University Hospital, Uchinada, Japan

**Keywords:** dry eye, depression, wheel-running, anxiety, mouse

## Abstract

Recent clinical studies indicate that dry eye is closely associated with psychiatric disorders such as depression and anxiety. Here, we investigated whether two types of mouse dry eye models showed depressive-like behavior in forced swim and sucrose preference tests, and whether voluntary wheel-running helped ameliorate depressive states. To reproduce the dry eye models, the exorbital lacrimal glands (ELG) or exorbital and intraorbital lacrimal glands (ELG+ILG) were bilaterally excised from male C57BL/6J mice. Tear volume was persistently reduced in both models, but the ELG+ILG excision mice exhibited more severe corneal damage than the ELG excision mice. In the forced swim and sucrose preference tests, the gland excision mice showed longer immobility and shorter climbing times, and lower sucrose preference than sham-operated mice, respectively, which appeared earlier in the ELG+ILG excision mice. Wheel-running activities were significantly lower in the ELG+ILG excision mice, but not in the ELG excision mice. After short-period wheel-running, the longer immobility times and the shorter climbing times in the forced swim completely disappeared in both models. Our results suggest that dry eyes might directly cause a depressive disorder that depends on the severity and duration of the ocular surface damage, and that voluntary motor activity could help recovery from a depressive state induced by dry eye.

## Introduction

Dry eye, one of the most common diseases of the ocular surface, manifests as tear film instability and hyperosmolarity of tears, which induces damage, inflammation, and sensory abnormality in the cornea and conjunctiva. Patients with dry eye disease have significant pain and reduced visuality, and often show limitations in performing daily activities, poor general health, and depression (Nelson et al., 2017). The severity of dry eye symptoms was revealed to be positively correlated with depression and anxiety; improvements to dry eye disease following ophthalmic treatments are associated with relief of depressive symptoms (Kobashi et al., 2018; Bitar et al., 2019). However, other eye diseases such as glaucoma (Lim et al., 2016) and macular degeneration (Jacob et al., 2017) are unlikely to show an association with depression and anxiety. Therefore, dry eye disease may cause significant stress and present a psychological burden on patients, and its treatment may alleviate these psychiatric disorders. Recently, a few studies using animal models of dry eye disease revealed a correlation with anxiety (Mecum et al., 2020; Fakih et al., 2021); however, the interaction between depression and dry eye disease has been less investigated.

Anxiety and depression show close associations with motor activity. Mice showing depressive-like behavior through chronic social defeat stress showed lower voluntary wheel-running activity, whereas voluntary wheel-running was more effective at improving depressive-like behavior than treatment with the antidepressant fluoxetine (Pagliusi et al., 2020). Acute treadmill running was shown to increase the c-fos-positive neurons containing serotonin and corticotropin-releasing factor in the dorsal raphe and hypothalamic paraventricular nucleus of mice, resulting in amelioration of anxiety-like and depressive-like behavior (Otsuka et al., 2016). In restraint-stressed rats, wheel-running improved anxiety-like behavior, depressive-like behavior, and memory impairment, and was accompanied by expression of brain-derived neurotrophic factor (BDNF) in the hippocampus (Lapmanee et al., 2017). In humans, acute aerobic exercise can improve mood and fatigue in patients with depressive and anxiety disorders (Bartholomew et al., 2005; Knapen et al., 2009). Therefore, exercise is thought to be an effective therapy for psychiatric disorders.

In this study, to clarify the relationships between dry eyes, depression, and motor activity, we investigated: (1) whether mice exposed to the bilateral excisions of the exorbital lacrimal glands (ELG) or exorbital and intraorbital lacrimal glands (ELG+ILG) show depressive-like behaviors in forced swim and sucrose preference tests and changes in motor activities in the open-field test and in the wheel-running apparatus, and (2) whether voluntary wheel-running ameliorates the depressive-like behaviors induced by the lacrimal gland excisions.

## Methods and materials

### Animals

Male 6–7-week-old C57BL/6J mice purchased from SLC (Shizuoka, Japan) were housed in clear acrylic cages (32 cm length × 21 cm width × 13 cm height) in a temperature (25 ± 1°C)- and humidity (45%)-controlled room with a 12 h light/dark cycle (lights on from 7 AM to 7 PM). They were habituated to these conditions for 1 week before the experiments. The cages were cleaned once a week, with cleaning being avoided within 24 h before a behavioral test. All animal procedures were approved by the Ethics Committees of Kanazawa Medical University (2020–26). Animals were treated humanely, in accordance with the Guiding Principles for the Care and Use of Laboratory Animals set by the Japanese Pharmacological Society. All studies involving animals are reported in accordance with the ARRIVE guidelines. A total of 160 animals were used in the experiments described in this report.

### Dry eye models

To reproduce aqueous-deficient dry eye models with different severities, 7–8-week-old mice were anesthetized with ketamine (90 mg/kg, i.p., Daiichi Sankyo, Tokyo, Japan) and xylazine (5 mg/kg, i.p., Zenoaq, Fukushima, Japan) and their bilateral exorbital lacrimal glands (ELGs) or bilateral ELGs and intraorbital lacrimal glands (ILGs) were removed according to the report by Shinomiya et al. (2018). The incisions were disinfected with 0.3% tobramycin (Nitto Medic Co., Toyama, Japan) and sutured. Sham-operated animals were treated with skin incisions and a drop of antibiotic in the surgical areas, and were then sutured without gland excision. The animals were equally allocated to the gland excision and sham-operation groups, with simple randomization for each experiment. Eight mice were allocated to each group in the behavioral tests. The mice received water and food *ad libitum* and were housed in a cage for 6 or 12 weeks after surgery. The mice in each group were housed in groups of two to four because individual housing can be stressful and cause depressive-like behavior. The researchers who assessed tear volume, fluorescein staining, and animal behaviors were blinded to the experimental groups to avoid subjective bias.

### Tissue staining

The tissues excised in the surgery were immersed in a cold fixative solution containing 4% paraformaldehyde in 0.1 M phosphate buffer (pH 7.4) for more than 24 h at 4°C. The specimens were then placed in 0.01 M phosphate-buffered saline containing 20% sucrose for 12 h for cryoprotection and were embedded in Tissue Tek (Sakura Finetek, Torrance, CA, USA). Sections of 10 μm were cut and stained with Mayer's hematoxylin (Merck, Darmstadt, Germany). Sections were mounted on slides in a mounting medium with a coverslip and were examined under a microscope.

### Measurement of tear volume and fluorescein staining

The volume of tear fluid was measured in both eyes with phenol red threads (Zone-Quick; AYUMI Pharmaceutical Co., Tokyo, Japan) placed under the lower lid for 30 s. The lower lid was pulled down slightly and the thread was gently placed onto the nasal palpebral conjunctiva. The thread was removed after 30 s and the length of the red-stained portion was measured to an accuracy of ± 0.1 mm using a vernier caliper and used as a measure of the tear volume in the conjunctival sac and tear secretion during the measurement.

To assess corneal damage, a fluorescein solution was prepared by dissolving 0.7 mg fluorescein (FLUORES^®^ AYUMI Pharmaceutical Co., Ltd., Tokyo, Japan) in 300 μL of sterile saline. Under an anesthetized condition with isoflurane (Pfizer, New York, NY, USA), 3 μL of fluorescein solution was applied to each eye. After superfluous solution was removed with KimWipes, green fluorescent illumination (infiltrated fluorescein) was examined with a blue LED excitation light (470 nm). The fluorescein infiltration score was categorized from 0 to 4 (0: no fluorescence, 1: fluorescence on <25% of the whole ocular surface, 2: fluorescence on <50%, 3: fluorescence on <75%, and 4: fluorescence on more than 75%) for each eye.

### Forced swim test

The forced swim test was performed according to previously reported methods (Yankelevitch-Yahav et al., 2015; Liu et al., 2019). The mice in their home cages were transported to a behavioral testing room (23°C ± 2°C) more than 1 h before the swim test, to allow acclimatization to the environment. Acrylic cylinders (30 cm height, 9.5 cm internal diameter) were filled with fresh tap water at 27°C ± 2°C to a water depth where the mice could not touch the bottom with their tails (approximately 15 cm). The temperature of the water gradually decreased during a test trial and was around 25°C at the end of the trial, which is the generally reported water temperature for a forced swim test. Each mouse was placed in the water filled cylinder for 6 min, and was removed immediately after the 6 min had elapsed. The behavior of the mice during the test was recorded with a video camera. Immobility, climbing and swimming over the last 4 min was observed by two trained observers who were blinded to the experimental groups. The values measured by the two observers were averaged and then used for statistical analysis. The immobility duration was quantified as that when a mouse floated motionless, including the movement necessary to keep its head above water. A significant extension of immobility duration and a significant reduction of climbing duration were considered to represent depressive-like behavior in this study.

### Sucrose preference test

The procedure for this test was based on methods described by Hashikawa et al. (2017). Animals were habituated to drinking water from two bottles for 2 days. In the sucrose preference test, two pre-weighed bottles (one containing tap water and the other containing a 2% [w/v] sucrose solution) were presented to each animal for 4 h. The position of the water and sucrose bottles (left or right) was switched every 2 h. After the 4 h, the bottles were re-weighed and the weight difference was taken to represent the animal's intake from each bottle. The sum of water plus sucrose intake was defined as the total intake, and sucrose preference was expressed as the percentage of sucrose intake relative to the total intake. A decrease in sucrose preference was considered to reflect a depressive state.

### Open-field test

The open-field test was performed to assess spontaneous locomotor activity and anxiety-like behavior, as described previously (Shuo et al., 2020). Mice were placed individually in a circular open-field chamber (diameter, 80 cm; height, 50 cm). All movements were recorded using a digital camera for 15 min and were tracked using SMART v 3.0 (Panlab Harvard Apparatus, Holliston, MA, USA). The floor was divided into outer and inner zones by a 40-cm diameter circle in the center of the field. The total distance traveled in the whole field and the amount of time spent in the inner zone were statistically analyzed. The apparatus was cleaned after each trial.

### Measurement of wheel-running activity

Six or 12 weeks after the surgery, wheel-running activities were measured for 3 days. Mice were individually housed in a cage (230 × 100 × 100 mm) with a running wheel (230 mm in diameter, 55 mm wide; Natsume Seisakusho, Tokyo, Japan), as previously described (Chen et al., 2008; He et al., 2020). These cages were maintained at 25°C ± 1°C under a 12 h light/dark cycle (lights on from 7 AM to 7 PM) with food and water available *ad libitum*. The rotations of the wheel from wheel-running activity were counted with an automatic counter and were recorded at 7 AM and 7 PM. The counting was started at 7 AM on the first day.

### Experimental design

Four experiments were performed using ELG and ELG+ILG excision mice and the sham-operated control mice, according to the following schedules. Each experiment was performed with different cohorts. All experiments were performed with 8 mice in each experimental group.

#### Experiment 1

**Figure 1:**
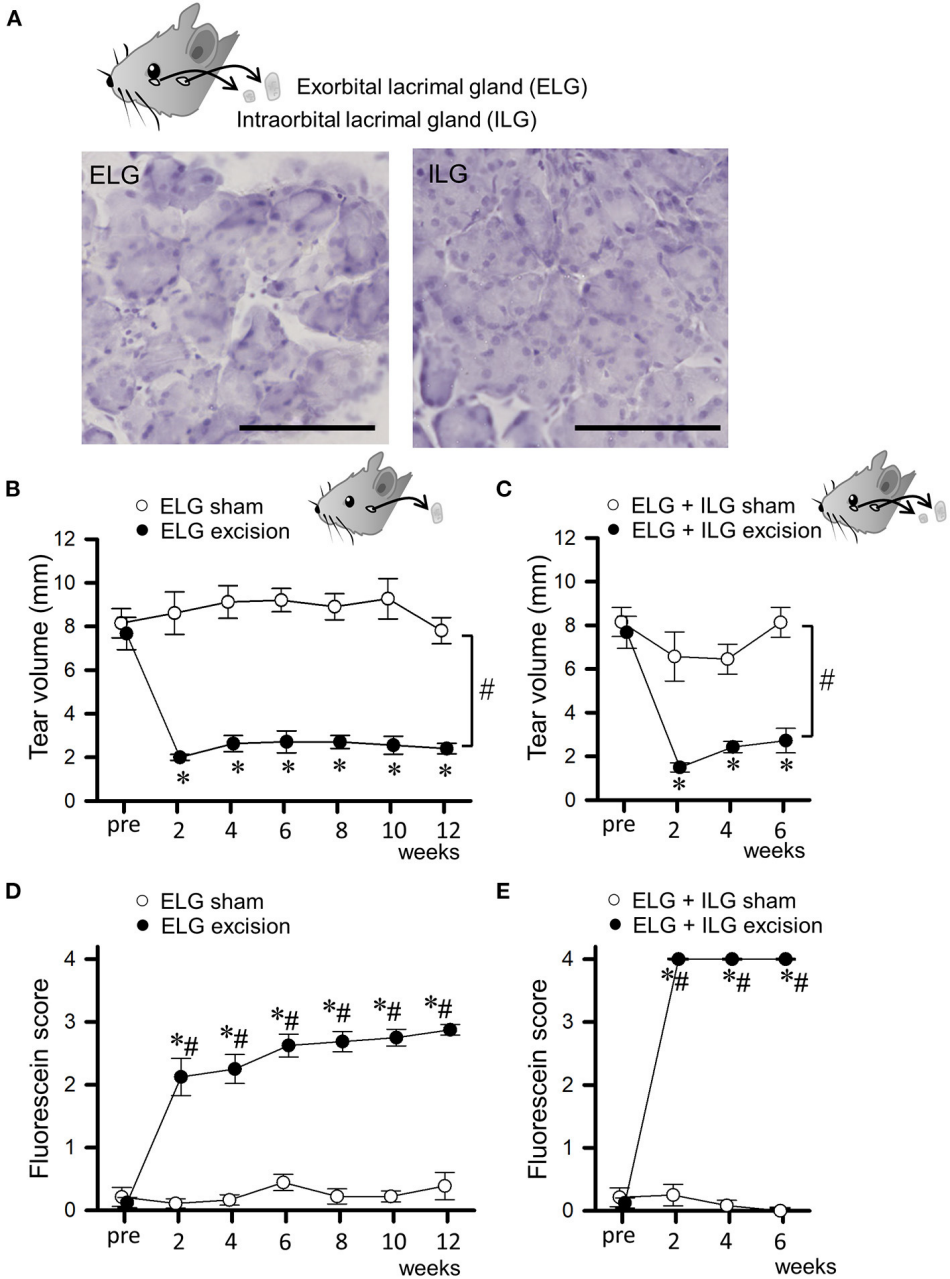
Changes in tear volume and fluorescein score after excision of lacrimal glands. **(A)** Microscopic images of the excised exorbital lacrimal glands (ELGs) and intraorbital lacrimal glands (ILG). ELGs and ILGs were excised from an 8-week-old C57BL/6J mouse and stained with hematoxylin. The horizontal bars represent 100 μm. **(B,C)** Tear volume measured with phenol red thread placed under the lower eye lid for 30 s. The mean lengths of the red-stained portion in ELG or ELG+ILG sham-operated (open circles), ELG excision [**(B)**, closed circles], and ELG+ILG excision mice [**(C)**, closed circles] are shown at each time point. **(D,E)** The mean fluorescein scores in ELG or ELG+ILG sham-operated (open circles), ELG excision [**(D)**, closed circles], and ELG+ILG excision mice [**(E)**, closed circles] are shown at each time point. Each point and the corresponding vertical bar represent the mean ± SEM (*n* = 8). *P < 0.05 *vs*. before the excision, #*P* < 0.05 *vs*. the sham-operated group.

Tear secretion and corneal damage were assessed every 2 weeks before and after surgery (Figure 1). Thirty-two mice divided into 4 experimental groups (ELG excision, ELG sham, ELG+ILG excision and ELG+ILG sham) were used in this experiment.

#### Experiment 2

**Figure 2:**
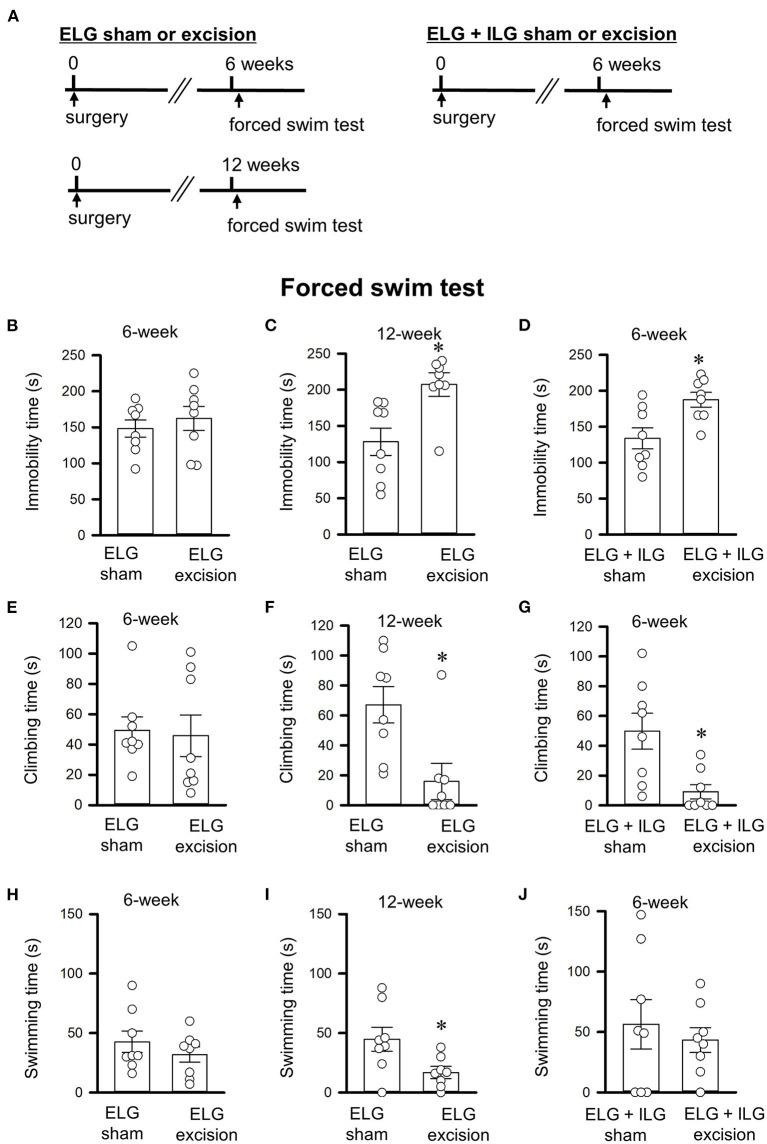
Effects of lacrimal gland excisions on behavioral performance in the forced swim tests. **(A)** Timelines of the experimental procedure. **(B–J)** Total times of immobility **(B–D)**, climbing **(E–G)**, and swimming **(H–J)** for 4 minute durations in the forced swim test. **(B,C,E,F,H,I)** Tests were performed on ELG excision and sham-operated mice at 6 weeks **(B,E,H)** and 12 weeks after surgery **(C,F,I)**. **(D,G,J)** Tests were performed on ELG+ILG excision and sham-operated mice at 6 weeks after surgery. Each column and the corresponding vertical bar represent the mean ± SEM (*n* = 8); open circles indicate individual values. *P < 0.05 *vs*. the sham-operated group.

The forced swim test was conducted 6 or 12 weeks after surgery (Figure 2). The behavioral data 12 weeks after surgery was obtained from mice without experiencing any behavioral tests 6 weeks after surgery, in order to avoid the effects of previous behavioral experiences. Forty-eight mice divided into 6 experimental groups (ELG excision and sham for the test at 6 weeks, ELG excision and sham for the test at 12 weeks, ELG+ILG excision and sham for the test at 6 weeks) were used.

#### Experiment 3

**Figure 3:**
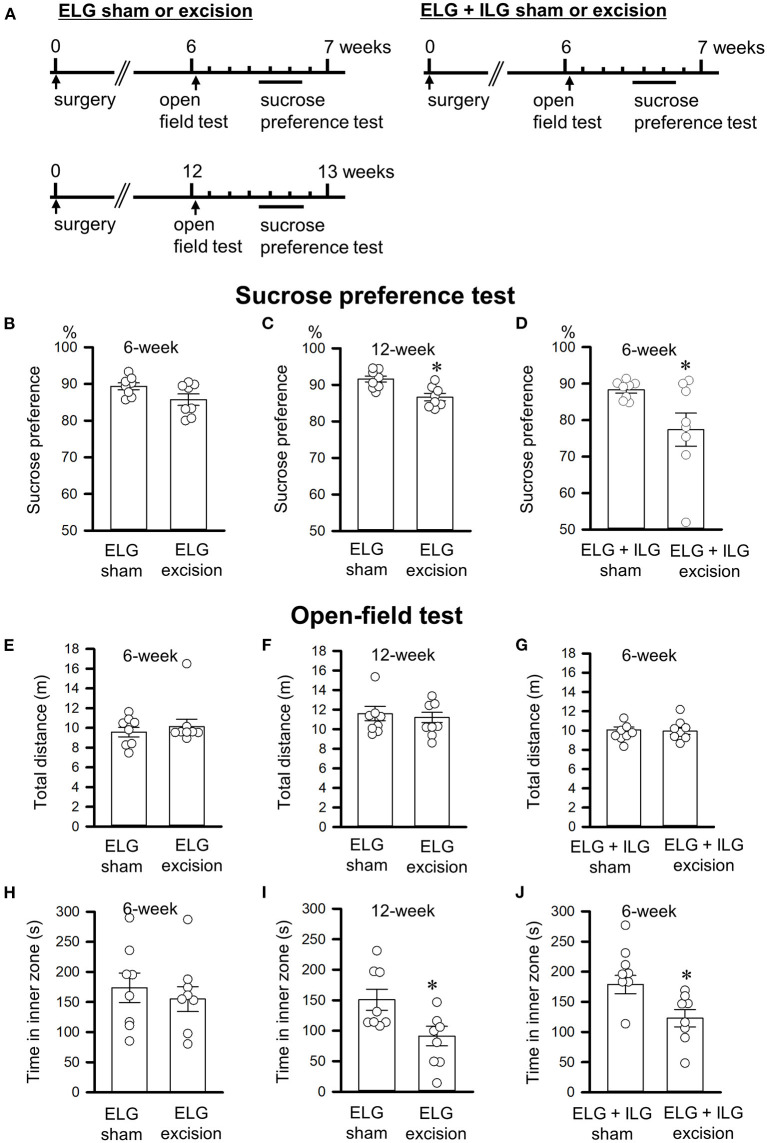
Effects of lacrimal gland excision on behavioral performance in the sucrose preference and open-field tests. **(A)** Timelines of the experimental procedure. **(B–D)** Percentage sucrose intake relative to total intake in the sucrose preference test. **(E–J)** The total distance in the whole field **(E–G)** and the time spent in the inner zone **(H–J)** in the open-field test. **(B,C,E,F,H,I)** Tests were performed on ELG excision and sham-operated mice at 6 weeks **(B,E,H)** and 12 weeks after surgery **(C,F,I)**. **(D,G,J)** Tests were performed on ELG+ILG excision and sham-operated mice at 6 weeks after surgery. Each column and the corresponding vertical bar represent the mean ± SEM (*n* = 8); open circles indicate individual values. *P < 0.05 *vs*. the sham-operated group.

The open-field test followed by the sucrose preference test were performed 6 or 12 weeks after surgery (Figure 3). Forty-eight mice divided into 6 experimental groups (ELG excision and sham for the tests at 6 weeks, ELG excision and sham for the tests at 12 weeks, ELG+ILG excision and sham for the tests at 6 weeks) were used.

#### Experiment 4

**Figure 4:**
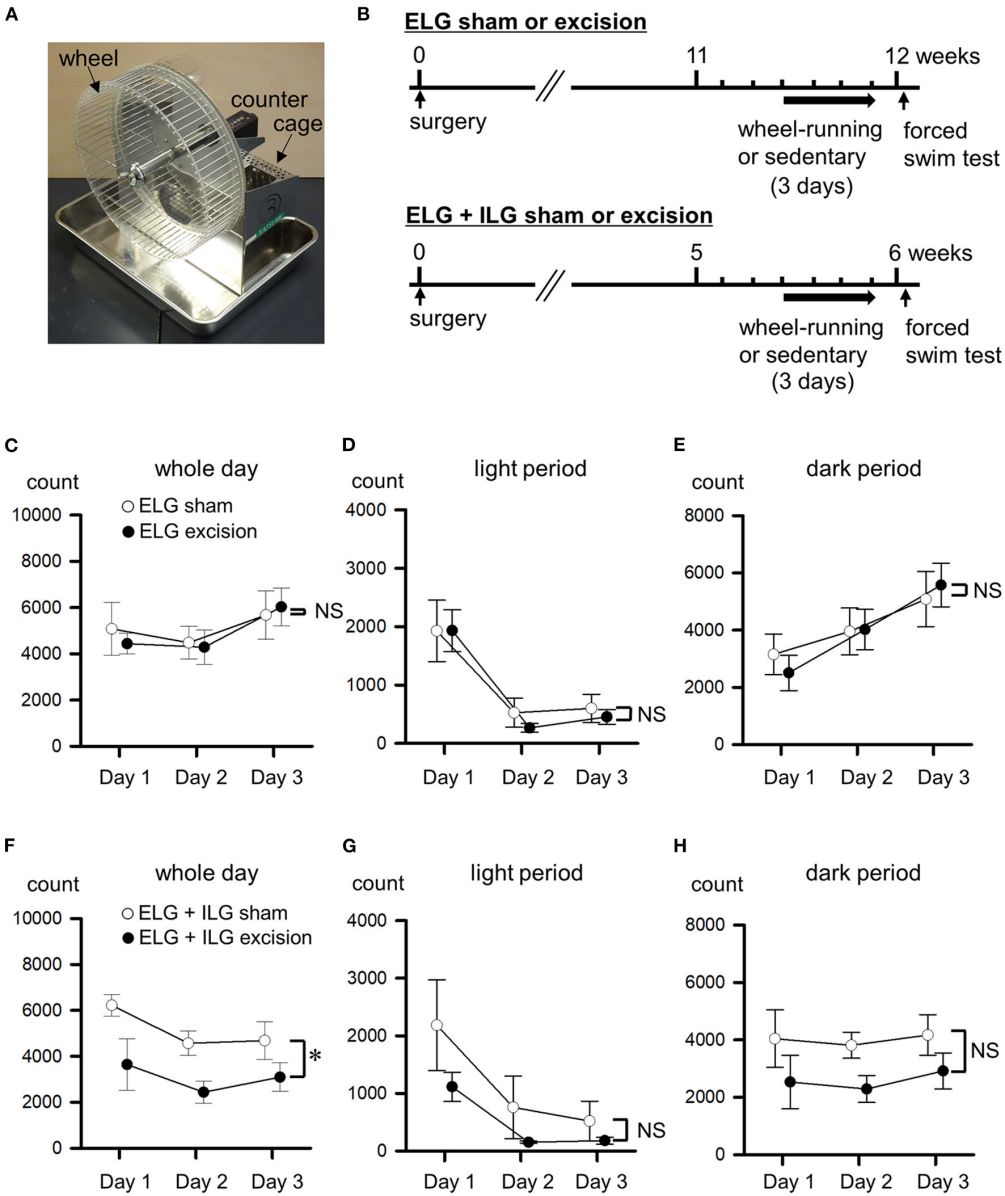
Effects of lacrimal gland excisions on wheel-running activity. **(A)** Running wheel apparatus. **(B)** Timelines of the experimental procedure. **(C–H)** The wheel-running activities from the first to third day are shown as counts of wheel rotation in a whole day **(C,F)** divided into the light period **(D,G)** and dark period **(E,H)**. Sham-operated mice (open circles), ELG excision mice [closed circles in **(C–E)**], and ELG+ILG excision mice [closed circles in **(F–H)**]. Each point and the corresponding vertical bar represent the mean ± SEM (*n* = 8). *P < 0.05 *vs*. the sham-operated group.

**Figure 5:**
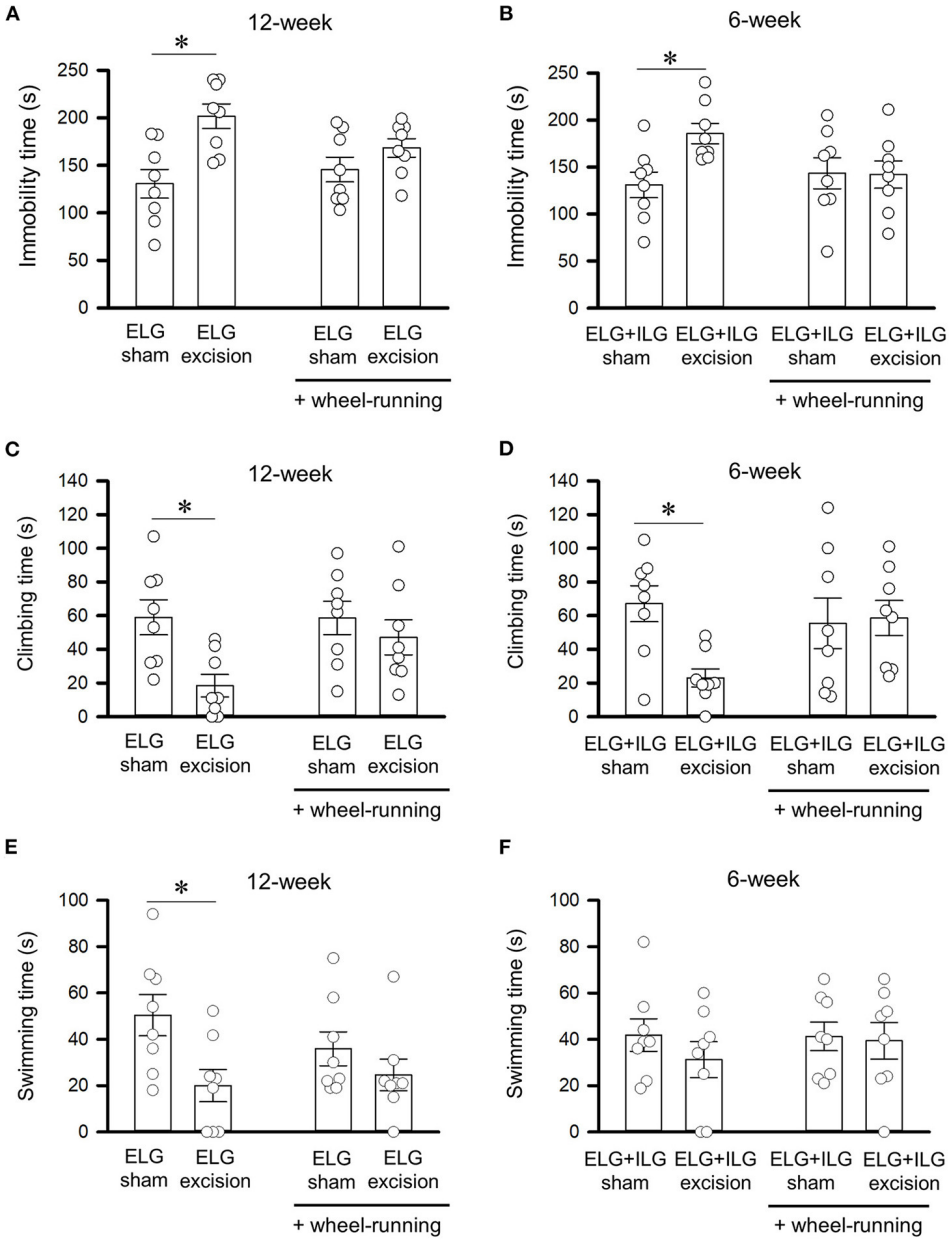
Effect of voluntary wheel-running on behavioral performance in the forced swim test. Total times of immobility **(A,B)**, climbing **(C,D)**, and swimming **(E,F)** for 4 min durations in the forced swim test were observed after wheel-running for 3 days, as shown in Figure 4B. Tests were performed with ELG excision and sham-operated mice at 12 weeks after surgery **(A,C,E)**, and with ELG+ILG excision and sham-operated mice at 6 weeks after surgery **(B,D,F)**. Each column and the corresponding vertical bar represent the mean ± SEM (*n* = 8); open circles indicate individual values. ^*^P < 0.05 *vs*. the sham-operated group.

The 3 day wheel-running evaluation was performed 11 weeks after ELG excision or the sham-operation, and 5 weeks after ELG+ILG excision or the sham-operation (Figure 4). Thereafter, the forced swim test was carried out (Figure 5). Thirty-two mice divided into 4 experimental groups (ELG excision, ELG sham, ELG+ILG excision and ELG+ILG sham) were used.

### Statistical analysis

Data were analyzed with SigmaPlot 13.0 software (Systat Software Inc., San Jose, CA, USA). Results are expressed as mean ± standard error of the mean (SEM). Unpaired Student's *t*-tests were used where assumptions of normality (Shapiro-Wilk test) and equal variance (Brown-Forsythe test) were met, with these being replaced by the Mann-Whitney test where appropriate. The results of the tear volume and wheel-running experiments were analyzed using two-way repeated measures analysis of variances (ANOVAs). Two-way ANOVAs were used for analyzing the effect of wheel-running on the behavioral changes in the forced swim tests. A *P* < 0.05 was considered statistically significant.

## Results

### Excision of lacrimal glands decreased tear secretion and damaged the corneal surface

We bilaterally excised only the ELGs, or both the ELGs and ILGs, in C57Bl/6J mice, and assessed their tear secretion and corneal damage every 2 weeks. Figure 1A shows microscopic images of the excised tissues stained with hematoxylin, with these tissues exhibiting the typical appearance of exocrine glands, confirming that the ELG and ILG were accurately excised. The tear volume measured with phenol red thread showed a substantial and sustained decrease after surgery in the ELG excision mice (closed circles in Figure 1B; F_1, 14_ = 183.88, *P* < 0.05, two-way repeated measures ANOVA with Dunnett test) and ELG+ILG excision mice (closed circles in Figure 1C; F_1, 14_ = 40.76, *P* < 0.05, two-way repeated measures ANOVA with Dunnett test), whereas the tear volume showed no difference in the ELG or ELG+ILG sham-operated mice (open circles in Figures 1B,C). The fluorescein staining revealed the development of corneal damage after surgery. The ELG excision mice showed moderate corneal damage and a gradually exacerbated corneal condition from 2 to 12 weeks post-operation (H = 28.15, *P* < 0.05, Kruskal-Wallis one-way ANOVA on ranks with Dunnett test; closed circles in Figure 1D), whereas the sham-operated mice showed no significant damage to the cornea (H = 3.613, *P* = 0.73). However, the ELG+ILG excision mice exhibited strong corneal damage immediately after the excision (H = 22.74, *P* < 0.05; closed circles in Figure 1E). Furthermore, the ELG+ILG excision group showed higher fluorescein scores than the ELG excision group from 2 to 6 weeks post-operation. Both mice models were aqueous-deficient dry eye models; the ELG+ILG excision mice had more severe damage on the ocular surface than the ELG excision mice.

### Lacrimal gland excision increased depressive-like behaviors in the forced swim and sucrose preference tests

The forced swim and sucrose preference tests were performed 6 or 12 weeks after gland excision in accordance with the schedules shown in Figures 2A, 3A. Extension of the immobility time and a decrease in the sucrose intake were assessed as a depressive-like behavior in this study. At 6 weeks post-operation, the ELG excision mice showed no significant difference in immobility time (t_14_ = −0.69, P = 0.50, Student's *t*-test; Figure 2B), climbing time (t_14_ = 0.22, *P* = 0.83; Figure 2E), or swimming time (t_14_ = 0.95, *P* = 0.36; Figure 2H) compared with the sham mice; however, the ELG+ILG excision mice that exhibited severe dry eye signs showed significantly longer immobility times (t_14_ = −2.98, *P* < 0.05, Student's *t*-test; Figure 2D) and shorter climbing times (t_14_ = 3.14, *P* < 0.05; Figure 2G). At 12 weeks post-surgery, the ELG excision mice also showed significantly increased immobility times (t_14_ = −3.18, *P* < 0.05, Student's *t*-test; Figure 2C) and decreased climbing times (t_14_ = 2.97, *P* < 0.05; Figure 2F) and swimming times (t_14_ = 2.48, *P* < 0.05; Figure 2I). Very similar results were observed in the sucrose preference test. Compared with the sham-operated mice, the ELG excision mice at 12 weeks (t_14_ = 3.57, *P* < 0.05, Student's *t*-test; Figure 3C) and the ELG+ILG excision mice at 6 weeks (t_14_ = 2.25, *P* < 0.05, Student's *t*-test; Figure 3D) showed a significantly lower percentage of sucrose intake, whereas the ELG excision mice at 6 weeks showed no significant difference (t_14_ = 2.01, *P* = 0.064, Student's *t*-test; Figure 3B). To reduce the number of animals used from the viewpoint of animal welfare, the effect of ELG+ILG excision on the behavioral tests was not examined at 12 weeks when the ELG excision mice showed a significant change in behavioral performance. Therefore, we suggest that the dry eye model mice developed depressive-like behaviors that depended on the severity and duration of their dry eyes.

### Lacrimal gland excision increased anxiety-like behavior without changing spontaneous locomotor activity in the open-field test

The open-field test was performed 6 or 12 weeks after gland excision (Figure 3A). The total distance traveled in the whole field and the time spent in the inner zone were evaluated as spontaneous locomotor activity and anxiety-like behavior, respectively. Excision of the lacrimal glands showed no significant effect on the total distance (t_14_ = −0.73, *P* = 0.48, Student's *t*-test, Figure 3E; t_14_ = 0.33, *P* = 0.75, Student's *t*-test, Figure 3F; t_14_ = −0.66, *P* = 0.52, Student's *t*-test, Figure 3G), which suggests that the excision had no effect on spontaneous locomotor activity. ELG excision at 6 weeks post-operation tended to result in a decrease in the time spent in the inner zone (t_14_ = 0.36, *P* = 0.73, Student's *t*-test; Figure 3H), while ELG excision mice at 12 weeks (t_14_ = 2.94, *P* < 0.05) and ELG+ILG excision mice at 6 weeks (t_14_ = 3.49, *P* < 0.05) spent significantly less time in the inner zone than sham-operated mice (Figures 3I,J). These results confirmed those in previous reports (Mecum et al., 2020; Fakih et al., 2021).

### Excision of both ELGs and ILGs decreased wheel-running activity

At the time when mice developed depressive-like behavior in the forced swim and sucrose preference tests, wheel-running activity was measured for 3 days (Figure 4B) using the apparatus shown in Figure 4A. This experiment was performed with different animals to those used for the results shown in Figures 1–3. Twelve weeks after the surgery, the ELG excision and sham-operated mice showed almost the same wheel-running activity in both the light period (F_1, 14_ = 0.20, P = 0.66, two-way repeated measures ANOVA; Figure 4D) and dark period (F_1, 14_ = 0.001, *P* = 0.97; Figure 4E), as well as over the whole day (F_1, 14_ = 0.01, *P* = 0.97; Figure 4C). Six weeks after excision, the ELG+ILG excision mice exhibited significantly lower wheel-running activity than the sham-operated mice over the whole day (F_1, 14_ = 6.28, *P* < 0.05, two-way repeated measures ANOVA with Dunnett test; Figure 4F), and tended to decrease their activity during the light period (F_1, 14_ = 4.56, *P* = 0.05; Figure 4G) and the dark period (F_1, 14_= 3.87, *P* = 0.078; Figure 4H). Therefore, the severe dry eye model mice might decrease their voluntary motor activity.

### Voluntary wheel-running ameliorates the extended immobility time in the forced swim test that was induced by gland excision

The day after the 3 day exposure to the running wheel apparatus, the ELG excision mice, ELG+ILG excision mice, and sham-operated mice were subjected to the forced swim test. The ELG excision mice showed a significant increase in immobility time (F_1, 31_ = 13.28, *P* < 0.05, two-way ANOVA with Dunnett test, Figure 5A) and significant decreases in climbing time (F_1, 31_ = 7.62, *P* < 0.05, Figure 5C) and swimming time (F_1, 31_ = 7.60, *P* < 0.05, Figure 5E), while treatment through wheel-running had no significant effect on immobility time (F_1, 31_ = 0.52, *P* = 0.48, Figure 5A), climbing time (F_1, 31_ = 2.26, *P* = 0.14, Figure 5C) and swimming time (F_1, 31_ = 0.43, *P* = 0.52, Figure 5E). Furthermore, the immobility, climbing and swimming times of the ELG excision mice after wheel-running was equivalent to those of the sham-operated mice at 12 weeks after surgery (*P* = 0.22, *P* = 0.40 and *P* = 0.30, respectively), although the ELG excision mice without wheel-running significantly extended the immobility time and significantly reduced the climbing and swimming times (*P* < 0.05; Figures 5A,C,E). ELG+ILG excision also significantly increased immobility time (F_1, 31_ = 4.62, *P* < 0.05, two-way ANOVA with Dunnett test, Figure 5B) and significantly decreased climbing time (F_1, 31_ = 4.71, *P* < 0.05, Figure 5D) but not swimming time (F_1, 31_ = 0.74, *P* = 0.40, Figure 5F), while wheel-running showed no significant change in immobility time (F_1, 31_ = 1.25, *P* = 0.27, Figure 5B), climbing time (F_1, 31_ = 1.20, *P* = 0.28, Figure 5D) and swimming time (F_1, 31_ = 0.27, *P* = 0.61, Figure 5F). The ELG+ILG excision mice at 6 weeks post-surgery also showed no significant change in immobility and climbing times after exposure to the wheel-running apparatus (*P* = 0.72 and *P* = 0.40, respectively; Figures 5B,D), whereas the ELG+ILG excision mice without wheel-running showed a significantly extended immobility and shortened climbing times (*P* < 0.05; Figures 5B,D). Therefore, voluntary wheel-running activities might ameliorate depressive-like behavior shown by the forced swim test.

## Discussion

We found that two dry eye mouse models with different severities exhibited depressive-like behaviors in mice undergoing the forced swim and sucrose preference tests, although the spontaneous locomotor activities in the open-field test were not changed. Hence, dry eye might be directly associated with depression, as well as the previously reported association with anxiety (Mecum et al., 2020; Fakih et al., 2021). In addition, severe dry eye induced by ELG+ILG excision caused depressive-like behavior in both tests within a shorter period (6 weeks) than ELG excision alone (12 weeks), and significant decreases in voluntary wheel-running activities that may correspond with depressive mood. Thus, high-severity dry eye might cause a faster onset of behavioral disorders related to depression than mild-severity dry eye. Our animal studies using aqueous-deficient dry eye models showed that chronic and severe dry eye can be an important risk factor for depression. It is important that our behavioral results are interpreted with caution because factors other than a depressive state could also be involved in the behavioral changes. Forced swim tests are often used to measure depressive states, but they can show false positive results that merely reflect the activation state of the animals. Actually, although the lacrimal gland excision mice showed a significant change in sucrose preference, the difference was small. In addition, a visual disturbance induced by ocular surface disorder would directly affect the performance of wheel-running, because the significant reduction of wheel-running activities was only observed when mice have the severest corneal damage. Further experiments with other behavioral tests such as social interaction tests are needed to confirm the presence of a depressive state.

Chronic ocular dryness induces inflammation of the ocular surface and subsequently alters the physiological properties of corneal nerves and their neuronal transmission in the brain, resulting in sensory abnormalities including severe pain (Kovács et al., 2016; Launay et al., 2016; Fakih et al., 2019; Li et al., 2019; Guerrero-Moreno et al., 2020; Masuoka et al., 2020). A chronic painful sensation including neuropathic pain has been reported to cause several mood disorders, such as anxiety and depression, with differences in psychiatric burden between each pain model causing variations in the latency of onset of depression (4–8 weeks) (Yalcin et al., 2011; Gambeta et al., 2018). The severe dry eye model used in the present study induced depression within 6 weeks; hence, dry eye strongly affects psychiatric function, similar to neuropathic pain from other sources. In addition to sensory abnormality on the ocular surface, other symptoms such as visual disturbance could also cause dry eye to have a strong impact on depression. In fact, a recent clinical study implied that dry eye patients with visual blurring are predisposed to depressive symptoms (Liyue et al., 2016).

According to Cochrane, while pharmacological and psychological therapies are effective for the treatment of depression in many studies, some national health service guidelines mention that exercise can be used as different treatment choice, although its effectiveness is inconclusive. In the present study, a short period of voluntary wheel-running ameliorated the depressive-like behavior in both dry eye models. Voluntary motor activities could be considered as side-effect-free therapeutics for dry eye patients with depression. Motor activities are reported to attenuate inflammatory and neuropathic pain in rodents. For instance, a voluntary wheel-running exercise in mice decreased persistent hyperalgesia induced by prostaglandin E_2_ acting on prostaglandin receptor coupled with TRPV1 (Sartori et al., 2020). A short period of treadmill running attenuated activation of microglia in the spinal cord caused by chronic constriction injury, and improved hyperalgesia in mice (Cobianchi et al., 2010). Relief from painful sensations through performing motor activities could ameliorate a depressive state in mice suffering from dry eye in the present study. On the other hand, motor activities are reported to directly improve depressive states in both clinical and animal research (Bartholomew et al., 2005; Knapen et al., 2009; Otsuka et al., 2016; Lapmanee et al., 2017; Pagliusi et al., 2020). However, 14–28 days were required to obtain the above-mentioned beneficial effects of exercise in these reports, whereas 3 days of exercise reversed the extension of immobility time and the reduction of climbing time in the forced swim test in the present study. The consecutive 3 days of exercise did not ameliorate the reduction in wheel-running activity in the ELG+ILG excision mice, and therefore the facilitation of physical activity after wheel-running could also affect our results, irrespective of the anti-depressive effect.

In conclusion, our findings show that (1) chronic ocular dryness caused a depressive state that depended on the severity and duration of ocular surface damage in mice, and (2) the voluntary wheel-running improved the dry eye-induced depressive-like behavior. Therefore, active treatment of dry eye would be important to avoid the risk of psychiatric disorder. In addition, voluntary exercise is expected to be an effective remedy to ameliorate and prevent depressive reactions caused by dry eye. Importantly, forced and voluntary exercise are reported to affect anxiety differently (Leasure and Jones, 2008); therefore, we will have to carefully consider the quality of exercise for treatment of depression caused by dry eye in the future.

## Data availability statement

The original contributions presented in the study are included in the article/supplementary material, further inquiries can be directed to the corresponding author.

## Ethics statement

The animal study was reviewed and approved by the Ethics Committees of Kanazawa Medical University.

## Author contributions

TM designed the study. TM, KN, HN, and QH performed research and analyzed data. TM, JU, TK, and TI wrote the manuscript reviewed by all authors. All authors contributed to the article and approved the submitted version.

## Funding

This work was supported by JSPS KAKENHI grants (19K07117, 19K09961, 19K18895, 20K09814, 20K22987, 22K16960, and 22K07358), Grant for Collaborative Research from Kanazawa Medical University (C2021-4 and C2022-2), and a grant from the Smoking Research Foundation of Japan (2021G008).

## Conflict of interest

The authors declare that the research was conducted in the absence of any commercial or financial relationships that could be construed as a potential conflict of interest.

## Publisher's note

All claims expressed in this article are solely those of the authors and do not necessarily represent those of their affiliated organizations, or those of the publisher, the editors and the reviewers. Any product that may be evaluated in this article, or claim that may be made by its manufacturer, is not guaranteed or endorsed by the publisher.
